# Wine & Brandy

**Published:** 1872-07

**Authors:** P. H. Hayes


### MEDICAL PATRONAGE OF WINE AND BRANDY.

P. H. HAYES, M. D.

It is now a settled thing that iron, and quinine, and cod liver oil, and opium, and bromide of potassium, and many other truly valuable drugs, are such only as they supply some abnormal want in the system. Thus in the terrible exhaustion which follows malarious chills, there is no substitute for quinine, and in the African and Syrian fevers of this type, not only is the enormous quantity of thirty to sixty grains given at a single dose without injury, but as an absolute necessity from impending death. In the simple bloodless condition from impaired assimilation, there is no perfect substitute for iron. Where there is a hot head, a flushed and burning face from active congestion, and too much combustion in the brain, bromide of potassium meets the immediate want. In bodily conditions attended by pain, what can we do without opium or some of its preparations ?—Again it has been proven that chloroform never did any harm upon the field of battle, where it was given to those already in pain from their wounds, as well as to prevent further pain during operations.

Now is it not absurd to give a man in health quinine, or opium, or iron, or chloroform ?—Can these or any other drugs make a sound man more sound ; can they make a strong man more strong, or a well man better ? Nay, but they will give a well man a headache, a fever. They will make him sick. There is no good in these things except as they do truly meet and satisfy a want, which some form of disease, or pain, or exhaustion has caused. Herein lies the whole gist of skill in medical practice, to have such exact knowledge of diseases, and the indications for treatment that the right medicine shall be chosen. Herein do sound doctors take sharp sight with a rifle and a single bullet, while others load a blunderbuss in hopes that some part of the load will hit the mark.

But what would you say of a man who should keep upon the street a shop for the sale of opium, or quinine, or chloroform, or corrosive sublimate, or nux vomica, upon the principle that these things are good for well people, and the crowd are to step in and buy at discretion?—When from exhaustion, or pain, or disease any one of these things is needed, then and *then only* is it *medicinal.* When not thus needed, these things are poisons.

Suppose it to be true that there are some states of exhaustion, bodily debility or infirmity, in which brandy or wine are to that state what quinine is to an ague or chloroform to a wounded man under a surgical operation. If you say brandy is good for a typhoid fever, or in other diseases of exhaustion, *as such*, why then give it to a well man? Will it make him better? No, but it will soon create in him a disease both bodily and mental, itself cannot cure, nor indeed is there any remedy but in total abstinance. Would it not then require the wisest judgment to tell whether in any given case a drug or stimulant would *cure* a disease or whether it would *create* a disease.

If the drift of my reasoning be true, brandy and wine should just as certainly be put under the care of physicians, to be prescribed alone by them, as that drugs should be, and for precisely the same reasons.

If shops for the sale of drugs for men and women to buy and use on their own judgment, and on the principle that they are good for well people, are absurd, it is for the same reason true of all forms of alcoholic stimulants.

Amid the great strifes and excitements of business, the nervous system is over-taxed and the digestion weakened, and men almost instinctively seek for compensation, and too often in stimulants rather than in rest and recreation. Again there is with a large number, a continual effort to get hold of something which shall give intensity if not intoxication to life, and these will often seek it in opiates or stimulants. If we admit that there are cases of disease and exhaustion wherein these things are needed, then they should be prescribed by a wise physician, who has also a deep sense of his moral responsibility to his patients.

Some men are largely animal; their moral sense is feeble, and their will so weak it cannot resist the appeals of appetite. Some men are born halt, or lame, or they have become so by accident. What should we think of a man who would put a stumbling block in the way which all but lame men could leap, but these were sure to fall thereon. But if such a man merits execration, why not the man who causes his brother to fall and be hurt in his moral nature. Shall we curse the man who would lay a trap for a lame man for gain, and have no voice against him who traps those who are lame in appetite and in moral sense? Is it not as patent as the light of day, that thousands fall into the vice of drinking and other vices, not because they are *bad*, but because they are *weak*, and this is their moral infirmity?

A well man who takes his wine daily, will find that he has stimulated his appetite beyond the need of nature, and he goes along the high road to apoplexy, gout, or Bright's disease. In weak digestion from old age, or other causes wine in its finer qualities may be prescribed, if ever, yet even here exhilarating society at the table, a new hope, a new inspiration in life, has often wrought wonders, and in a more perfectly normal manner. A gentleman was invariably seized with distress and cramps in his stomach, after the meals he took at the table with his wife, with whom, I am sorry to say, he was on very bad terms, but never in town where he dined with a party of boon companions.

We are glad to see that in England the use of alcoholic stimulants has received a fresh discussion, and that names of the greatest worth in the profession record their protest against the immensely exaggerated value of these things as an element of diet, or as a supplementary aid to nutrition. They are two-edged swords cutting both ways. Too often are physicians tempted to prescribe them, from considerations touching their reputation, or because such prescriptions are so popular, and how often does the patient continue his prescription long after the occasion is past for which it is prescribed. How much mischief has thus resulted, it would be impossible to estimate.

I cannot close this article without mentioning a class of men, found in every community, who do the very coolest thing yet. They will keep and retail liquor in their shops and saloons, to every one who can be tempted to buy, but they will never touch a drop themselves. They have seen the ruin it works, and they do so thoroughly believe their eyes that for themselves they touch not and taste not. They *know* that wine is a mocker, that strong drink is raging, and that whosoever is deceived thereby is not wise. They are often fine-looking, well-dressed, well-barbered and well kept men. They are apparently good husbands and fathers, yet will they coolly ruin those who come within the power of their trade, and so thoroughly do they know this that they cannot be tempted themselves, nor will they associate for a moment with those they are ruining, nor will they let wife or child come near the devilish influence of this calling. These are temperence men with a witness to it.

